# Prediction of potential occurrence of historical objects with defensive function in Slovakia using machine learning approach

**DOI:** 10.1038/s41598-024-82290-1

**Published:** 2024-12-05

**Authors:** Jana Vojteková, Saeid Janizadeh, Matej Vojtek, Anna Tirpáková, Matej Ruttkay, František Petrovič

**Affiliations:** 1 Department of Geography, Geoinformatics and Regional Development, Faculty of Natural Sciences and Informatics, Constantine the Philosopher University in Nitra, Nitra, Slovakia; 2 Department of Civil, Environmental and Construction Engineering and Water Resources Research Center, University of Hawai’i at Manoa, Honolulu, HI 96822 USA; 3 Institute of Geography, Slovak Academy of Sciences, Bratislava, Slovakia; 4 Department of Mathematics, Faculty of Natural Sciences and Informatics, Constantine the Philosopher University in Nitra, Nitra, Slovakia; 5 Department of School Education, Faculty of Humanities, Tomas Bata University in Zlín, Zlín, Czech Republic; 6 Institute of Archaeology, Slovak Academy of Sciences, Bratislava, Slovakia; 7 Department of Ecology and Environmental Science, Faculty of Natural Sciences and Informatics, Constantine the Philosopher University in Nitra, Nitra, Slovakia

**Keywords:** Historical object with defensive function, Machine learning, Prediction, Slovakia, Environmental sciences, Scientific data

## Abstract

In this article, we aim at the prediction of possible locations of already defunct historical objects with a defensive function (HODFs) in Slovakia, which have not been found and documented so far, using three machine learning methods. Specifically, we used the support vector machine, k-nearest neighbors, and random forest algorithms, which were trained based on the following five factors influencing the possible occurrence of HODFs: elevation, distance from a river, distance from a settlement, lithological rock type, and type of representative geoecosystems. Training and testing datasets were based on a database of already documented 605 HODFs, which were divided into 70% of training samples and 30% of testing samples. All of the three models reached the AUC-ROC value over 0.74 based on the testing dataset. The best performance was recorded by the random forest predictive model with the AUC-ROC value equal to 0.79. The results of the random forest model were also validated with the recently documented HODFs via the archeological research.

**Supplementary Information:**

The online version contains supplementary material available at 10.1038/s41598-024-82290-1.

## Introduction

Currently, there is a need for interdisciplinary archeological-geographical research in revealing new historical objects and structures in landscape. For this purpose, geographers can provide various types of analyses and predictive models based on geospatial and remote sensing data ^1,2^.

Various types of contact and non-contact methods are used for the needs of detailed archaeological research. However, there is often a need to combine these two types of methods ^3^. As for the non-contact methods, the aerial imagery is the most accessible. However, field research combined with aerial photography cannot always provide archaeologists with sufficiently accurate data for their work. A number of archaeological sites are located in places covered with vegetation, which has a stabilizing effect on the archaeological remains. Forest and shrub cover can protect archaeological objects from the influence of erosion processes as well as other factors. For this reason, these areas are often of interest to archaeologists. Aerial photography in areas covered with vegetation can only detect objects of larger dimensions, and therefore it is necessary to use other more effective methods in such areas ^4^.

Another approach that is increasingly used in geoscience as well as in other research fields are machine learning methods. Machine learning algorithms found their usage in various fields of study, for example, in medicine, economics, speech recognition, and they are increasingly being used in the study of the Earth and landscape processes ^5–8^.

Machine learning (ML) is considered a part of artificial intelligence since it is the study of computer algorithms that automatically improve based on experience and use of data. ML algorithms create models based on sample data, also referred to as training data, on the basis of which the model learns to predict or make decisions without explicit programming ^9^. The most frequently used machine learning approach is the supervised learning, where the computer contains examples of inputs and required outputs. Supervised learning is not solely about learning rules, but it is about capturing patterns in data to make accurate predictions. The essence lies in extracting meaningful relationships between inputs and outputs rather than adhering strictly to predefined rules. It is about understanding the underlying structure of the data to generalize well to unseen instances. In this study, supervised machine learning models were employed to yield valuable insights for the identification and prediction of potential locations of HODFs in Slovakia. This contribution aids in heritage conservation and archaeological research endeavors. The second most used approach is unsupervised learning, where the learning algorithm itself is left to find structures in the inputs ^10^.

ML, i.e. data-driven models, may include for example, artificial neural networks (ANN), adaptive neuro-fuzzy inference systems (ANFIS), support vector machines (SVM), random forest (RF), Bayesian network (BN), decision trees (DT), and others ^11^. Several recent studies, focusing on the archaeological research, document the increasing use of machine or deep learning algorithms in this field ^12–17^. Furthermore, the combination of the machine learning method and analysis of LiDAR images can also help to discover the extinct archaeological monuments. This is also confirmed by the research of Albrecht et al. ^18^, who examined archaeological artifacts based on data from Mexico, where archaeologists had already done extensive fieldwork, manually recognizing and mapping local features, etc. They used this data as training and testing classifiers for deep learning. Oonk and Spijkerpre ^19^ used the machine learning method to classify archaeological soils. Similarly, Zhao ^20^ utilized machine learning techniques called CaffeNet and deep convolutional neural network for identification of immovable cultural relics in the historic city of Macau. Also, Yuan and McKee ^21^ highlighted the importance of using machine learning and remote sensing techniques for common geographical-archaeological research. Their study focused on an archaeological site in the Indian village of Huff. Besides that, Fernandez-Diaz et al. ^22^, Witharana et al. ^23^, Ninfo et al. ^24^ or Balsi et al. ^25^ focused on the importance of LIDAR mapping in geographical-archaeological research.

In recent years, ML methods have become increasingly prevalent in various fields of spatial modeling. These approaches provide opportunities for revealing valuable insights within complex and extensive datasets ^26^. In this context, random forest (RF), k-nearest neighbors (KNN), and support vector machine (SVM) models have proven effective in spatial modeling to various phenomena ^27–29^.

RF model displays adaptability in handling diverse response variables and mitigates bias towards correlated predictors through techniques like bootstrap aggregating and feature randomization ^30,31^. KNN, a non-parametric classification algorithm, makes predictions based on the majority class of the k-nearest neighbors of a given data point. It is known for its simplicity and effectiveness in modeling complex relationships in data ^32^. SVM is a powerful supervised learning algorithm, particularly effective in high-dimensional spaces and excels in finding the optimal hyperplane that separates data points into different classes with the maximum margin ^33^. Consequently, RF, KNN, and SVM models may offer promising avenues for spatial modeling, providing valuable insights for identifying and predicting the locations of potential HODFs in Slovakia.

Many remains of fortified settlements in Slovakia have evolved into a critical state through the time and there is a real threat that they may disappear in few years. Therefore, more and more attention is now being paid to their research in Slovakia, as documented, for example, by the works of Cheben and Daňová ^34^, Beljak Pažinová and Beljak ^35^, Slamova et al. ^36^ or Greif and Vlčko ^37^. HODFs are represented by objects like castles, fortresses, mansions, strongholds, watchtowers, city walls, summer castles, fortified churches, fortified mansions, citadels, guard bastions, fortified courtyards. These objects had a fortified structure, usually made of stone, which primary role was to protect the local people from potential invaders. Most of the HODFs were built in Middle Ages and in case of castles or mansions they represented also fortified residence of a king or nobility and their servants, with not only the defensive (border defense, roads) function, but also residential, administrative, control, representative, economic, or symbolic function ^38^.

In 2017, the Geodesy, Cartography and Cadastre Authority of the Slovak Republic started a project of aerial laser scanning (ALS) of the territory of the Slovak Republic with the aim of creating a new digital elevation model (DEM). The ALS DEM was finished for the whole territory of Slovakia in 2023. One of the practical applications of the ALS DEM is the creation of specialized visualizations of ALS raster products, enabling the detection of elements that are “invisible” by standard forms of visualization. Such elements include, for example, vanished mounds, roads, hillforts, and historical landscape structures ^39^.

For instance, based on the ALS DEM, archeologists from Slovakia revealed a unique roundel object from the end of Late Stone Age (around the middle of the 5th millennium BC) in the cadastral area of the Podhájska municipality. The presence of large-scale trench formations was determined using a field survey, LiDAR measurements, and a detailed geophysical survey.

The aim of this article is the analysis of potential occurrence of HODFs in Slovakia, which have not been found and documented so far, using three machine learning algorithms: SVM, KNN and RF. Based on the work of Vojteková et al. ^40^, the following factors were taken into the consideration: elevation, distance from a river, distance from a settlement, lithological rock type, and type of representative geoecosystem. Besides the testing dataset, the obtained results were validated against the newly documented HODFs by archaeological field research. This study tries to fill in the gap in archaeological-geographical interdisciplinary research, which exists in a national-scale mapping of potential occurrence of HODFs, which have been hidden so far to the archeologists. It is very difficult to find such objects in landscape although it happens from time to time, often by accident. This study attempts to reduce the extent where a possible HODF may exist and increase its chance to be found. Such task may be performed by the use of powerful ML models and robust training/testing data, which have not been so far used to tackle the problem of defining the areas with potential occurrence of HODFs.

## Research area

**Fig. 1:**
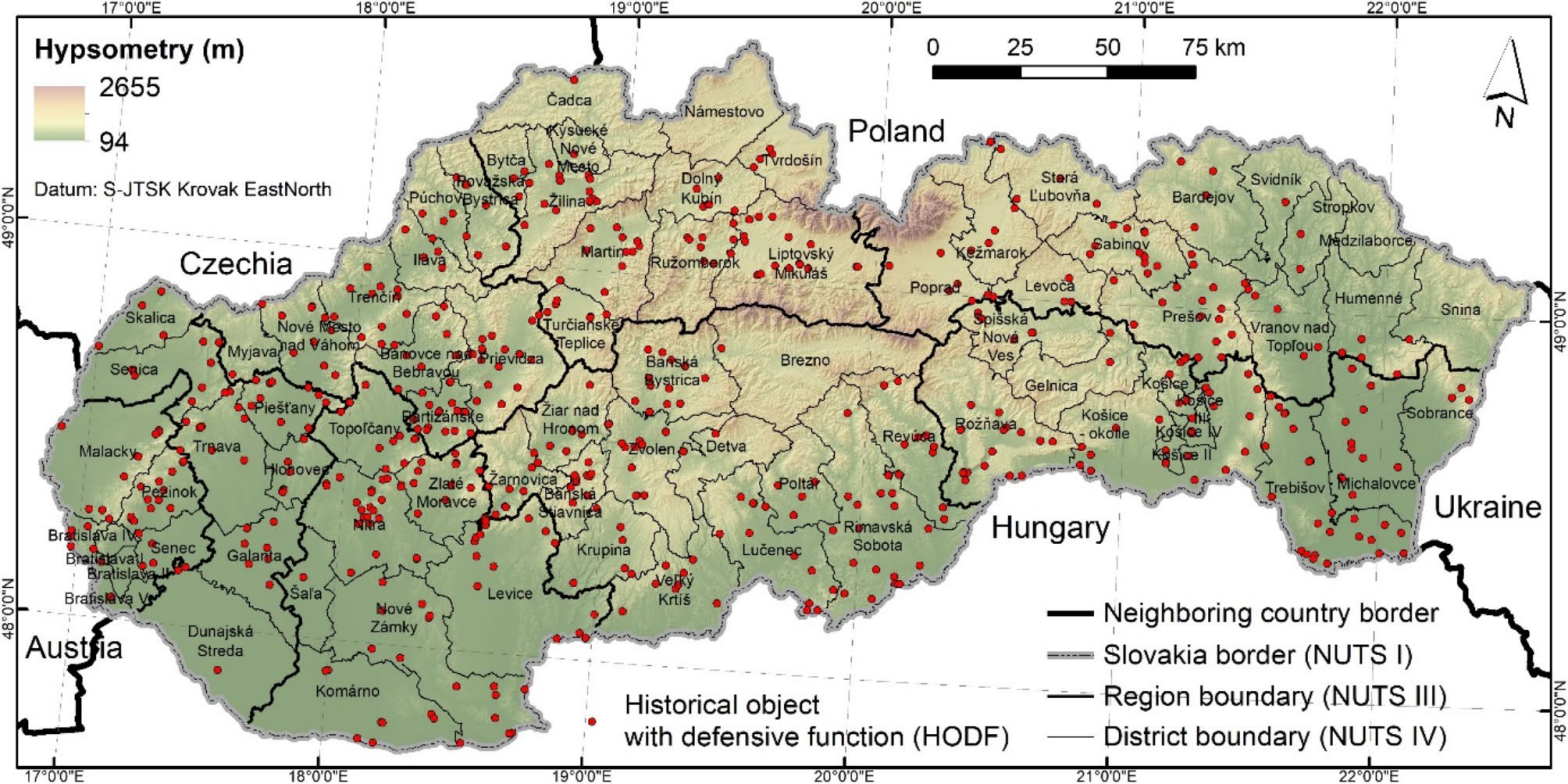
Research area and location of documented HODFs. This figure was generated in ArcGIS 10.2.2 software (https://www.esri.com/en-us/home).

The research area includes the whole territory of Slovakia with the extent of 49,035 km^2^. We focused on the potential occurrence of defunct historical objects that had a defense function (HODFs) in Slovakia. Figure 1 presents the distribution of 605 HODFs, like castles, fortresses, mansions, strongholds, watchtowers, city walls, summer castles, fortified churches, fortified mansions, citadels, guard bastions, fortified courtyards, which were have already been documented. The database of 605 HODFs was later used for training and testing phases of machine learning modeling (Fig. 1).

## Data and methods

### Data

**Fig. 2:**
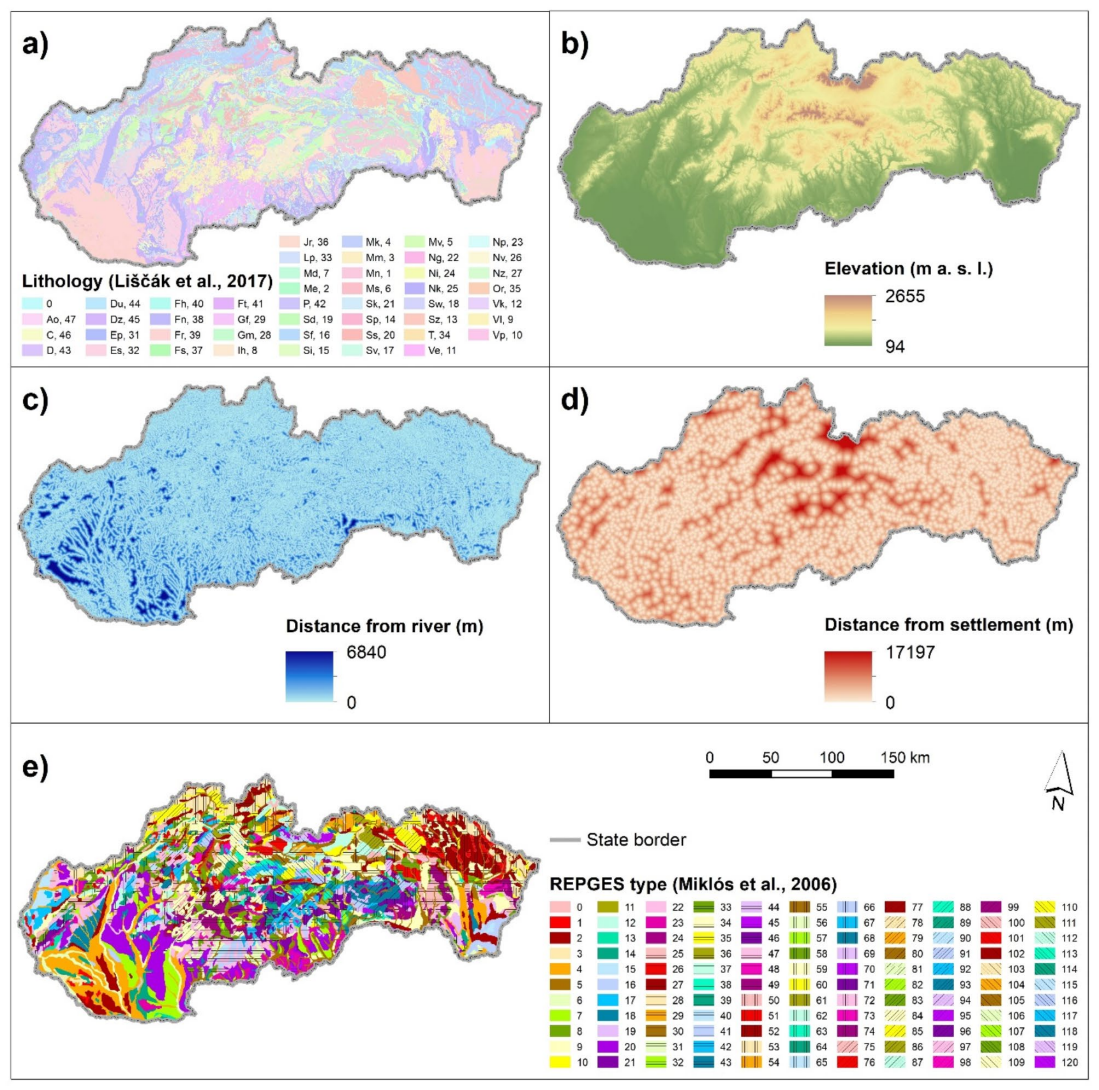
Factors used for modeling: (**a**) lithological rock type (see Liščák et al. ^37^ for detailed legend), (**b**) elevation, (**c**) distance from a river, (**d**) distance from a settlement, and (**e**) REPGES type (see Miklós et al. ^38^ for detailed legend). This figure was generated in ArcGIS 10.2.2 software (https://www.esri.com/en-us/home).

Altogether five factors: lithology, elevation, distance from a river, distance from a settlement, and type of representative geoecosystem (REPGES) were used in this study affecting the potential occurrence of HODFs (Fig. 2). The reason for selecting the five mentioned factors is that they all had certain effect on the environment where the HODFs were built in the past as well as on the conditions for living in them. The elevation factor, especially, higher elevated hills were most often selected as they had an influence on how the guards were able to view the surroundings of the HODF and face the attacks of enemy. Similarly, the river represented a difficult obstacle to overcome for enemies. Furthermore, the geological bedrock and its permeability as well as different terrain forms (i.e. REPGES types), such as rock cliffs, affected not only the place for constructing the HODFs, but also the actual conditions for living. In case of the settlements, for example, they were usually built close to the HODFs as they provided protection for local people in case of attacks of enemies ^36,37^. All factors were processed in ArcGIS 10.2.2 software (https://www.esri.com/en-us/home).

Lithology (i.e. lithological rock types) factor (Fig. 2a) was derived from the vector layer of Map of Engineering Geological Zones at a scale of 1:50,000 ^41^, which is publicly available through the map portal of the State Geological Institute of Dionýz Štúr.

Elevation factor (Fig. 2b) was derived from the digital elevation model (DEM) called DMR3.5, which is a hydrologically correct DEM with 10 m resolution. This DEM is publicly available through the website of the Geodetic and Cartographic Institute.

Factor of distance from a river (Fig. 2c) was created based on the river network vector layer obtained from the Basic Database for Geographic Information System (ZBGIS). This layer is harmonized with the DMR3.5 used in this study and is publicly available through the website of Geodetic and Cartographic Institute. The map of distance from a river was computed with the use of the Euclidean Distance tool in ArcGIS 10.2.2 software (https://www.esri.com/en-us/home).

Factor of distance from a settlement (Fig. 2d) was derived from vector layer of the centers of settlements obtained from the Geodetic and Cartographic Institute. We used again the Euclidean Distance tool in ArcGIS 10.2.2 software (https://www.esri.com/en-us/home) to create the map.

Factor of type of representative geoecosystems (REPGES) (Fig. 2e) was based on the publication by Miklós et al. ^42^. It represents landscape units, which are characterized by a certain diversity of conditions – different geological bedrock, morphometric, climatic, hydrologic, and soil conditions, which further condition the occurrence of different forms of ecosystems and biota. Based on the combination of azonal and zonal conditions, 120 types of REPGES were determined in Slovakia. This factor was derived from the vector GIS layer of REPGES types.

**Fig. 3:**
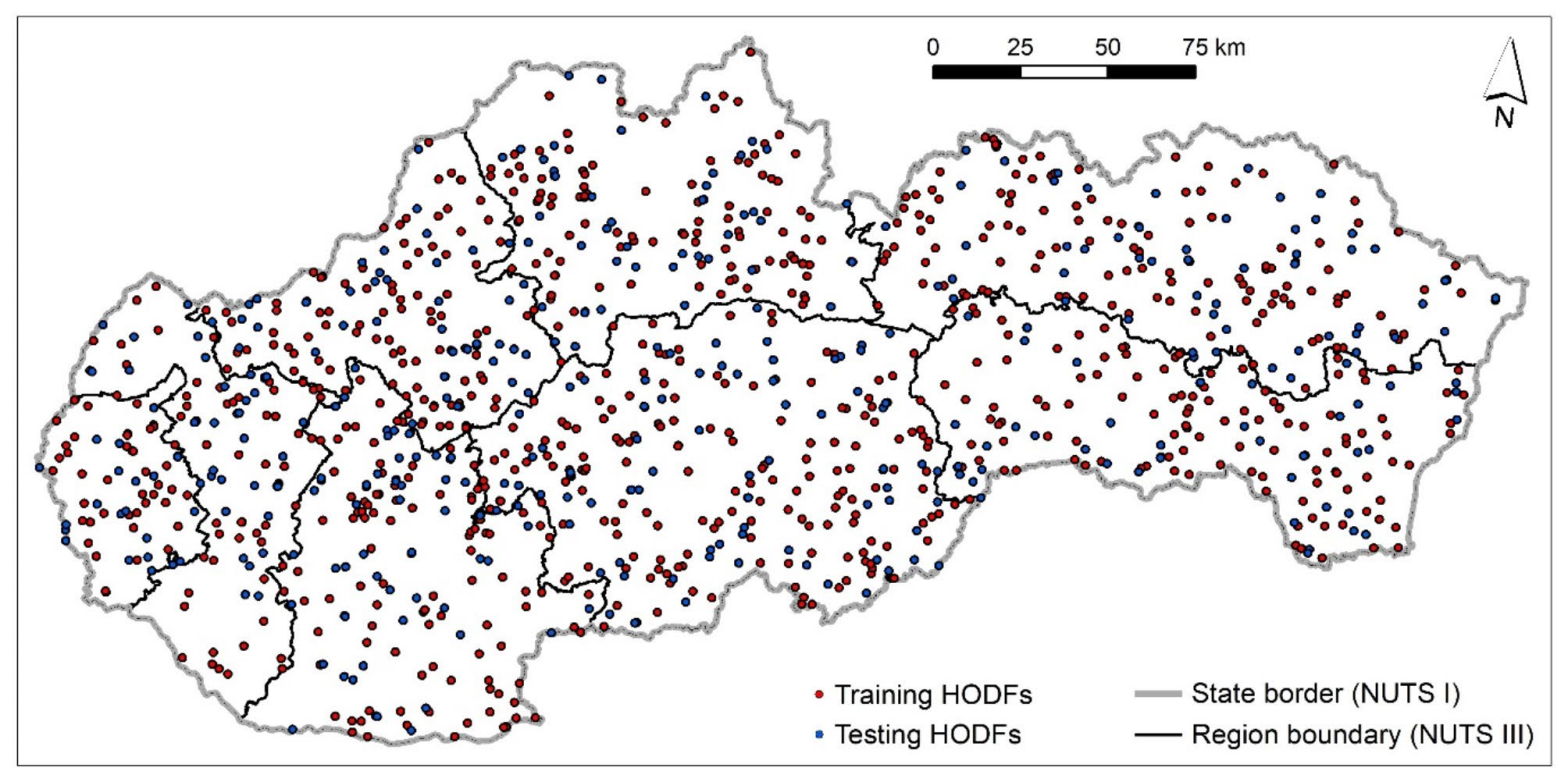
Distribution of training and testing points for ML modeling. This figure was generated in ArcGIS 10.2.2 software (https://www.esri.com/en-us/home).

The training dataset consisted of 846 training points, of which 423 points were randomly selected from the HODFs database. Another 423 points were randomly selected as non-HODF locations. The testing dataset contained 364 points. It was divided into 182 randomly selected points from the HODF database and another 182 randomly selected points, which represented the non-HODF locations (Fig. 3).

All of the previously mentioned factors were processed in ArcGIS 10.2.2 software (https://www.esri.com/en-us/home) and converted into a raster format with 50 m resolution due to computational demands with regard to the extent of the research area.

### Methods

#### Support vector machine (SVM)

Support vector machine (SVM) is a supervised machine learning algorithm used for classification and regression problems. It is based on the idea of finding the optimal hyperplane that maximally separates the samples into different classes. In SVM, the samples are represented as points in a high-dimensional feature space, and the goal is to find the optimal hyperplane that separates the samples into different classes while maximizing the margin between the classes. The margin is the distance between the hyperplane and the closest samples, known as support vectors, and it represents the generalization error of the model ^43,44^.

The SVM algorithm can handle both linear and non-linear problems by using a technique called kernel trick, which maps the original feature space into a higher-dimensional space where the samples can be separated by a hyperplane. SVM is a powerful algorithm that is widely used for classification and regression problems due to its ability to handle complex and non-linear relationships between the features and target variable. However, it is computationally expensive and can be sensitive to the choice of kernel and hyperparameters ^44,45^.

#### K-nearest neighbors (KNN)

K-nearest neighbors (KNN) is a popular machine learning algorithm used for classification and regression problems. It is based on the idea of the proximity of similar data points having similar outcomes. The algorithm starts by representing the training data as a set of points in a multi-dimensional feature space. Each sample is then assigned to one of several predefined classes. When a new sample is encountered, the algorithm identifies the KNN from the training data and predicts the class label of the sample based on the majority class among the KNN ^46,47^.

The value of K can be chosen using cross-validation and it determines the number of nearest neighbors that the KNN algorithm should consider. In general, a smaller value of K results in a more complex model, while a larger value of K results in a simpler model. KNN is a simple and fast algorithm that can be applied to both continuous and categorical data. It has the advantage of being non-parametric, meaning that it does not make any assumptions about the functional form of the relationship between the features and target variable. However, as the number of samples increases, the time required to find the nearest neighbors can become computationally expensive ^47,48^.

#### Random forest (RF)

Random forest (RF) is a popular machine learning algorithm that is used for both classification and regression problems. It is an ensemble learning method, which means it creates multiple decision trees, each trained on a random subset of the input data, and combines their predictions to produce a final output. The algorithm is highly effective in handling high-dimensional data and is robust to outliers and irrelevant features ^49^.

In other words, the RF algorithm classifies a new data point by combining the predictions of all decision trees in the forest, and selecting the class label that occurs most frequently. The RF algorithm is a versatile and powerful tool that is widely used in many applications, such as image classification, natural language processing, and recommendation systems. The algorithm is relatively simple to implement and has good performance compared to other machine learning algorithms ^49,50^.

#### Validation methods

Receiver Operating Characteristic (ROC) curve is a commonly used evaluation metric for binary classification problems in ML. It plots the True Positive Rate (Sensitivity) against the False Positive Rate (1—Specificity) for different thresholds used to make binary predictions. The Area Under the Curve (AUC) of the ROC curve is a single scalar value that summarizes the overall performance of the classifier ^51,52^. The AUC ranges from 0 to 1, where an AUC of 1 indicates a perfect classifier, while an AUC of 0.5 indicates a random classifier that performs no better than chance. An AUC greater than 0.5 indicates a better-than-random classifier, while an AUC less than 0.5 indicates a worse-than-random classifier ^53,54^.

Positive Predictive Value (PPV) and Negative Predictive Value (NPV) are evaluation metrics that measure the accuracy of positive and negative predictions, respectively. The ROC curve, AUC, PPV, NPV, Sensitivity, and Specificity are important evaluation metrics for binary classification problems in machine learning. They provide a comprehensive assessment of the classifier’s performance, taking into account both the accuracy of positive and negative predictions and the trade-off between them ^52,54^.

## Results

### Distribution of HODFs in factor classes

**Fig. 4:**
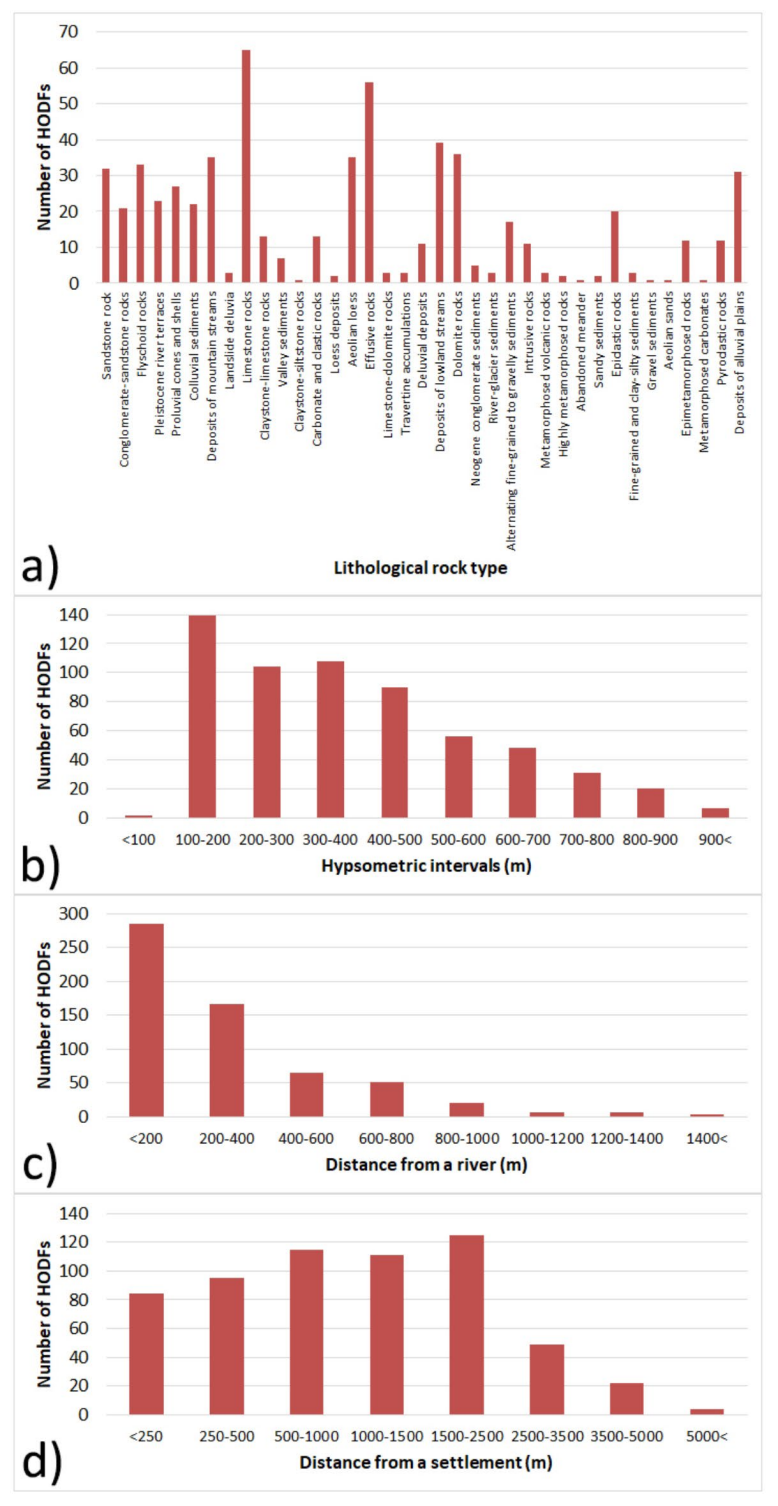
Number of HODFs in factor classes: (**a**) lithological rocky types, (**b**) hypsometric intervals, (**c**) distance from a river, and (**d**) distance from a settlement.

Based on Fig. 4a, we can see that the highest number of HODFs is included in the lithological rock type of limestone rocks (65 HODFs), followed by the effusive rocks (56 HODFs), and deposits of lowland streams (39 HODFs). Only 1 HODF belongs to the following lithological types: metamorphosed carbonates, aeolian sands, gravel sediments, abandoned meander, and claystone-siltstone rocks. As for the inclusion of HODFs in different hypsometric intervals (Fig. 4b), we recorded the highest number of HODFs in the interval 100–200 m (139 HODFs), followed by the interval 300–400 m (108 HODFs), and interval 200–300 m (104 HODFs).

As shown in Fig. 4c, the highest number of HODFs (284) is located in the distance of less than 200 m from a river and 167 HODFs belong the distance interval of 200–400 m from a river. On the other hand, only 4 HODFs are located within longer distance from a river of more than 1400 m. Figure 4d shows the number of HODFs as respect to their distance from a settlement. The interval 1500–2500 m includes the highest number of HODFs (125), followed by the interval 500–1000 m (115 HODFs) and 1000–1500 m (111 HODFs). The least HODFs (4) are located at a distance of more than 5000 m from a settlement. The number of HODFs in individual types of REPGES is presented in supplementary Table S1. Out of 120 REPGES types, HODFs were located in 70 of them. The highest number of HODFs (55) were included in the REPGES type 4—river floodplain in lowlands, followed by the REPGES type 5—river floodplain in basin or mountain valley with 51 HODFs, and REPGES type 6—fragmented meander plain with 37 HODFs.

### Potential sites of HODFs

**Fig. 5:**
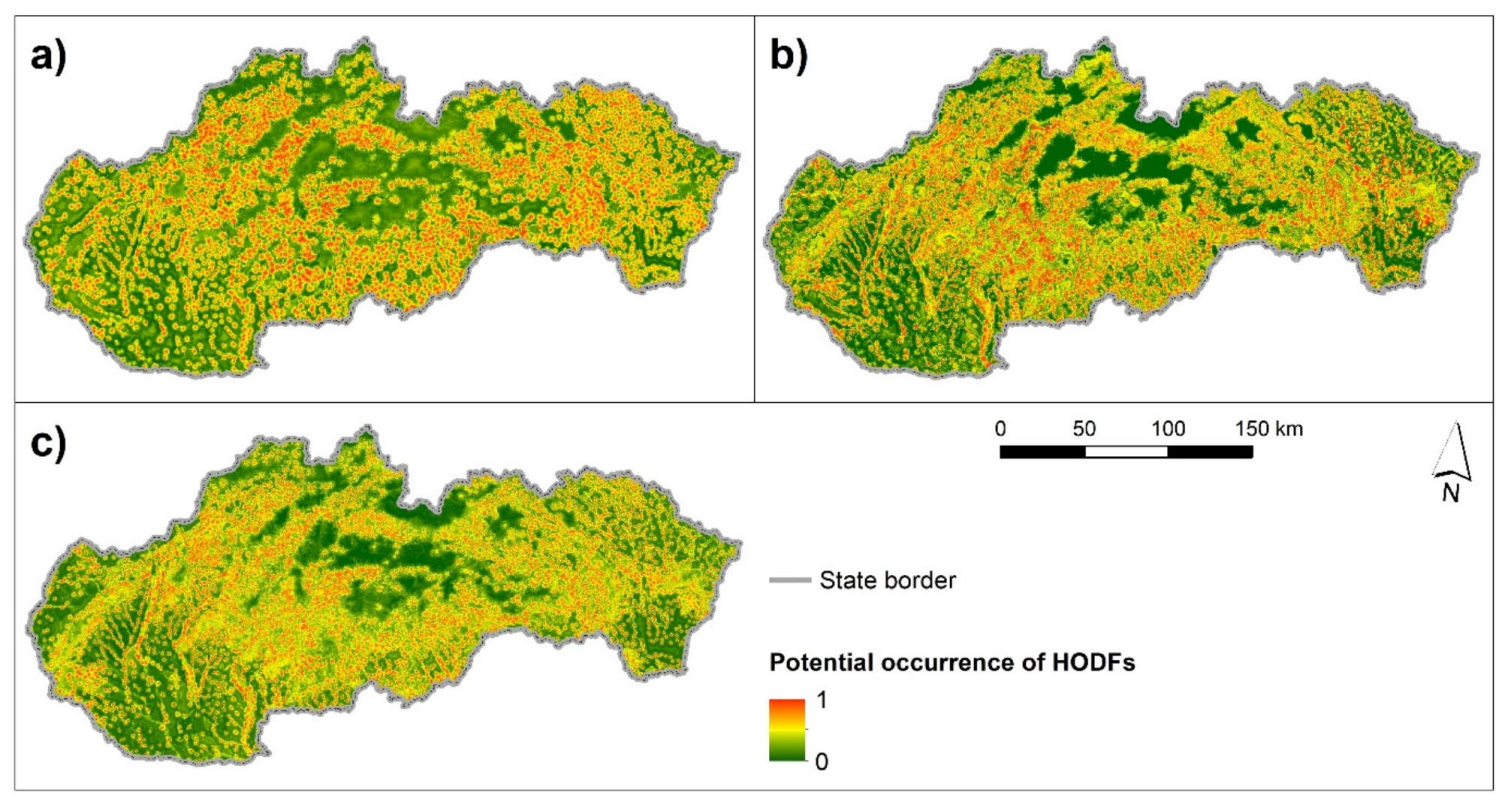
Potential occurrence of HODFs using ML models: (**a**) SVM, (**b**) KNN, and (**c**) RF. This figure was generated in ArcGIS 10.2.2 software (https://www.esri.com/en-us/home).

The maps in Fig. 5 present potential sites of occurrence of HODFs on a scale from 0 to 1, where 1 means the highest potential. The resulting map of the SVM model is presented in Fig. 5a, while the resulting KNN and RF models are presented in Fig. 5b and Fig. 5c, respectively.

### Accuracy evaluation of ML models

In order to evaluate the accuracy of ML models used in this study, five different metrics in training and testing stages were applied, which are shown in Table 1. In the training stage, the SVM and KNN models had comparatively lower accuracy than RF model. The AUC metric for training stage showed the following values for SVM, KNN, and RF models, respectively: 0.83, 0.94, and 0.99.

**Table 1:** Evaluation of models in training and testing stages.

Models	Stage	Metrics				
		Sensitivity	Specificity	PPV	NPV	AUC
SVM	Training	0.73	0.76	0.75	0.74	0.83
	Testing	0.66	0.69	0.69	0.66	0.74
KNN	Training	0.85	0.84	0.86	0.85	0.94
	Testing	0.71	0.67	0.69	0.69	0.76
RF	Training	0.96	0.97	0.96	0.98	0.99
	Testing	0.69	0.76	0.75	0.70	0.79

**Fig. 6:**
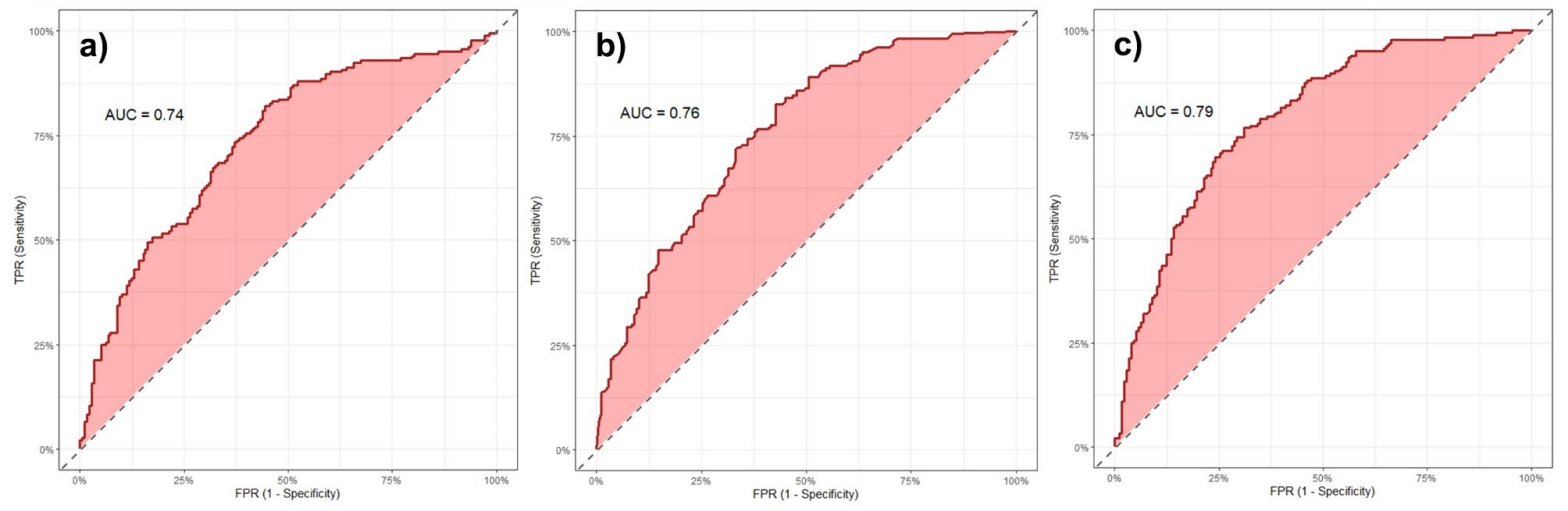
ROC-AUC in the testing stage of three ML algorithms used in this study: (**a**) SVM, (**b**) KNN, and (**c**) RF.

The results of accuracy evaluation of the selected ML algorithms based on the AUC for the testing stage are shown in Fig. 6. In the testing stage, all three ML algorithms performed in the interval between AUC = 0.7 and AUC = 0.8. The highest AUC value was recorded by the RF model (AUC = 0.79), followed by the KNN model (AUC = 0.76) and SVM model (AUC = 0.74).

### Factor importance analysis

The results of factor importance analysis for modeling of the potential occurrence of HODFs based on three ML algorithms are shown in Table 2. In this study, permutation importance (PI) was utilized to assess the importance of variables in KNN, SVM, and RF models. This method involves randomly permuting the values of each predictor variable and evaluating the model’s performance using the permuted data. The drop in performance after permuting a variable indicates its importance; the larger the drop, the more significant the variable ^55,56^. This analysis showed that each of the independent factors has various effects on the modeling of potential occurrence of HODFs sites using selected ML algorithms. The most important factor regarding all of the models was the distance from settlement, which was followed by the elevation factor. As for, the SVM model, the third most important factor was the REPGES type, followed by the factor of distance from a river. In case of the KNN model, the situation was similar as in SVM regarding the third and fourth most important factors. However, in case of the RF model, the third most important factor was distance from a river followed by the REPGES type. The lithology factor had the lowest importance in all of the models used.

**Table 2:** Results of factor importance analysis based on three ML algorithms.

Factor	SVM	KNN	RF
Elevation	26.46	22.12	38.71
Distance from a settlement	100.00	100.00	100.00
Distance from a river	2.13	1.79	15.37
Lithology	0.00	0.00	0.00
REPGES type	3.88	4.72	1.08

## Discussion

From a physical-geographical point of view, the locations of possible occurrence of HODFs were predicted mainly in the area of lowlands and hills, in valleys and along the watercourses.

By analyzing the results, we found that no documented HODF was located in five districts of Slovakia (Bratislava II, Bratislava III, Košice III, Námestovo, and Medzilaborce) and there was only one HODF in six districts (Bratislava I, Šaľa, Čadca, Kysucké Nové Mesto, Brezno, Snina, Svidník, and Košice II). However, using the ML modeling, possible locations of occurrence of HODFs were predicted in all of the districts in Slovakia. The largest number of HODFs (19–24), based on the previous research by Vojteková et al. ^40^, was located in the districts of Trebišov, Košice – okolie, Nitra, Liptovský Mikuláš, and Rimavská Sobota. Based on the results of this study, the highest number of predicted locations with possible occurrence of HODFs was in the districts of Košice – okolie, Rimavská Sobota, Michalovce, Vranov nad Topľou, and Humenné. On the other hand, the lowest number of locations with possible occurrence of HODFs was predicted by the RF model in the districts of Košice III and Bratislava I.

From the point of view of regional comparison, the highest number of documented HODFs was identified in the Banská Bystrica region, followed by the regions of Košice, Nitra, Trenčín, Žilina, Prešov, Trnava, and Bratislava with the smallest number of HODFs ^40^. As for the predicted occurrence of HODFs using the RF model, most of the locations fall within the Banská Bystrica region, followed by the regions of Košice, Prešov, Nitra, Trenčín, Trnava, and Žilina. The least locations were predicted in the Bratislava region.

As part of validation of the results, we verified some of the potential sites of HODFs by comparing them with the newly discovered archaeological sites as well as field survey. When comparing newly discovered archaeological sites, that were not included in our database of documented HODFs, with the predicted locations, we focused only on the results of the Random forest model, as it performed most accurately among the three ML models used. For comparison, we used the interval 0.9–1.0, which is considered to represent the highest potential for the occurrence of HODFs.

**Fig. 7:**
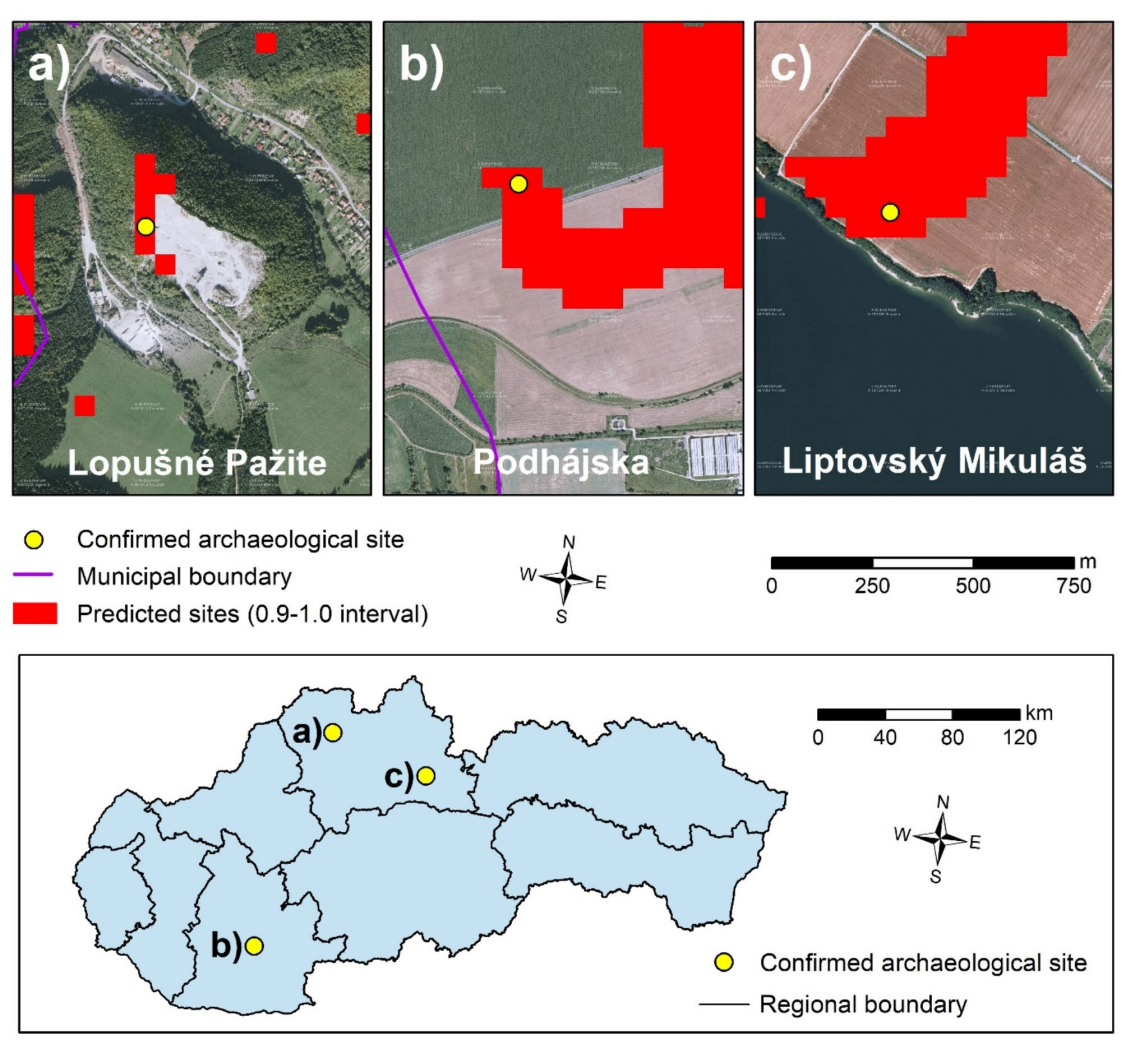
Examples of match between the newly discovered archaeological sites and predicted locations of HODFs by the RF model (pixels in interval 0.9–1.0): (**a**) castle in the Lopušné Pažite municipality, (**b**) roundel in the Podhájska municipality, (**c**) prehistoric settlement in Liptovský Mikuláš town. This figure was generated in ArcGIS 10.2.2 software (https://www.esri.com/en-us/home). Imagery source was represented by orthophotos freely available via the Web Map Service (WMS): http://tiles.geop.sazp.sk/base/service?.

We found several examples of newly documented HODF locations, which were not part of our HODF database, but were accurately modeled by the RF model. For example, the castle in the municipality of Lopušné Pažite was accurately predicted at the given location by the RF model (Fig. 7a). Although the castle was already discovered during the years 1978–1980, it was not included in our HODF database. It is assumed that it served the residents of the settlement as a refuge in case of impending danger. Archaeological research and finds date this location to the younger Iron Age and the settlement with the adjacent castle of smaller dimensions was used by people of the Púchov culture ^57^.

Furthermore, the RF model accurately predicted the roundel discovered in 2022 in the Podhájska municipality (Fig. 7b). Although this cult object from the end of the Early Stone Age is not fully considered a HODF, it probably served as a kind of calendar. The central part of the discovered cult object was an almost circular moat with a diameter of approximately 75 m and four entrances from roughly each side of the world. It was followed on the outside by a system of other ditches and palisades. This resulted in an almost square structure with slightly rounded walls and an external diameter of approximately 130 × 130 m. Another accurately predicted HODF, in terms of its location, was in the town of Liptovský Mikuláš, where the remains of a prehistoric settlement from the Bronze Age were discovered at the end of 2022 (Fig. 7c).

Despite the fact that the archaeological sites in the Podhájska municipality and Liptovský Mikuláš town do not show apparent signs to be HODFs, the RF model predicted both of them as suitable locations for the construction of a HODF. It means that ML can be helpful not only in the identification of HODFs, but also other historical locations that could have been inhabited or various constructions could have been located there in the past. Therefore, in further research, the cooperation between different experts, but especially, archaeologists and geographers would be appropriate to survey different parts of Slovakia, but mainly the eastern or central part of Slovakia, where a number of possible HODF locations were identified by the RF model and, currently, only some of them are documented.

Polla et al. ^58^ also point to the importance of involving various experts (archaeologist, historian, art historian, architect, etc.) as well as geographers in the research of historical objects as such. The means of historical work should not be only the analysis of stratigraphy in an archaeological probe or the profiling of architectural elements. Standard procedures applied when working with historical sources should remain part of the researcher’s equipment: inductive and deductive, direct and indirect, progressive and retrospective, geographical, philological or comparative method. The choice of individual methods is mainly conditioned by the concept of the research, as well as the scope and nature of the available sources. In addition to the accumulation of facts, the emphasis on their constant critical evaluation does not lose relevance. Despite the long-term tradition of forming methodological and interpretive equipment, the research of HODFs in practice shows that the history presented on the basis of written reports is an important, but far from the only area of knowledge and research ^38^.

However, predicting the potential occurrence of historical objects with defensive function in Slovakia using machine learning models offers several advantages despite its limitations. One notable limitation is the challenge of obtaining high-quality, comprehensive datasets, which can lead to inaccuracies in the models. Nonetheless, the findings can help identify regions that are particularly likely to contain such historical structures, highlighting potential archaeological hotspots. This information is critical for prioritizing preservation and research efforts and allows for comparisons across different regions, facilitating the identification of patterns and sharing of best practices for historical conservation. A human-in-the-loop approach^55–59^ may significantly help to clean and re-organize noisy datasets, thereby addressing this limitation and enhancing the accuracy and effectiveness of machine learning models by incorporating expert knowledge and iterative feedback for an empowered ML design experience.

Another problem that may potentially arise in ML modeling is overfitting. Overfitting occurs when a model is trained too well on the training data, but it performs poorly on unseen or new data. In other words, a high error rate in the testing data indicates overfitting. Following the common practice in literature, we used the hold-out method (i.e. split the dataset into two sets: training and testing). In our study, the model performs well not only on the training set, but also on the testing set. The results indicate good generalization capability on both the training and testing set. Furthermore, this study used the bootstrap method to fine-tune parameters in the KNN, SVM, and RF models, which is a technique known for mitigating overfitting, reducing variance, and enhancing algorithm stability by generating bootstrap samples from the training data based on caret package in R software ^60,61^.

## Conclusion

In this study, we used three machine learning algorithms to predict the potential sites for the occurrence of defunct HODFs, which have not been discovered and documented so far. Among the three ML models, the best performance was achieved by the RF model with the AUC-ROC value of 0.79 based on the testing dataset. Using the results of this model, we verified some of the predicted locations of potential occurrence of HODFs with the recently documented archaeological sites, such as previously mentioned sites in Podhájska, Lopušné Pažite or Liptovský Mikuláš. In these cases, the exact location was predicted by the RF model, which points to the effective usage of ML models, especially, as a primary method for identifying possible locations for the following detailed archaeological field research.

This work also points to the fact that although the HODFs are mainly studied from the historical and archaeological point of view, the modern trend is in a holistic and comprehensive approach, which is based on the participation of different fields of study and application of advanced geospatial technologies (LiDAR, drones, aerial photogrammetry, and so on) to the research of HODFs. The rationale for the useful application of ML methods and geospatial technologies in the research of HODFs is based on the assumption that HODF is an element in landscape, which changes in space and time.

## Electronic supplementary material

Below is the link to the electronic supplementary material.

Supplementary Material 1

## Data Availability

The input data presented in this study are freely available in Mendeley Data repository at 10.17632/k6mszh3rgc.2. Other requests on data used in this study can be sent to the corresponding author. The code used in this study is available via repository and released under the MIT license. It can be accessed at https://github.com/Saeidjanizadeh161/Historical-Objects-Modeling.
