# Supplementary material for: Prediction of potential occurrence of historical objects with defensive function in Slovakia using machine learning approach
Source: Sci Rep. 2024 Dec 5;14:30350. doi: 10.1038/s41598-024-82290-1 (PMC11621328; doi:10.1038/s41598-024-82290-1)
Supplement: Supplementary file 1 — Supplementary Material 1 [file 41598_2024_82290_MOESM1_ESM.docx]

**Supplementary Material**

Table S1. Number of HODFs in types of REPGES.

| **Types of REPGES** | **Number of HODFs** | **Types of REPGES** | **Number of HODFs** |
| --- | --- | --- | --- |
| 2 (lowland or basin depression) | 5 | 48 (rugged volcanic highlands – oak-hornbeam forests) | 2 |
| 4 (river floodplain in lowlands) | 55 | 49 (rugged volcanic highlands – oak-beech forests) | 24 |
| 5 (river floodplain in basin or mountain valley) | 51 | 51 (rugged flysch highlands – oak-hornbeam forests) | 4 |
| 6 (fragmented meander plain) | 37 | 52 (rugged flysch highlands – oak-beech forests) | 5 |
| 7 (river terrace or proluvial cone – oak-cerium forests) | 10 | 53 (rugged flysch highlands – beech forests) | 10 |
| 9 (river terrace or proluvial cone – oak-hornbeam forests) | 26 | 54 (rugged flysch highlands – beech-fir forests) | 1 |
| 10 (river terrace or proluvial cone – oak-beech forests) | 6 | 55 (rugged flysch highlands –fir-spruce forests) | 2 |
| 12 (river terrace or proluvial cone – fir-spruce forests) | 5 | 56 (rugged karst highlands – oak-cerium forests) | 2 |
| 13 (river terrace or proluvial cone) | 3 | 57 (rugged karst highlands – oak-hornbeam forests) | 4 |
| 14 (plain /dune plain or loess cover/ – oak-cerium forests) | 1 | 58 (rugged karst highlands – beech forests) | 7 |
| 15 (plain /dune plain or loess cover/ – oak forests) | 2 | 59 (rugged karst highlands – beech-fir forests) | 3 |
| 16 (plain /dune plain or loess cover/) | 4 | 60 (rugged highlands on variegated Mesozoic rocks – oak-cerium forests) | 6 |
| 18 (loess plateau – oak-cerium forests) | 10 | 62 (rugged highlands on variegated Mesozoic rocks – oak-hornbeam forests) | 18 |
| 19 (loess plateau – oak-hornbeam forests) | 1 | 64 (rugged highlands on variegated Mesozoic rocks – beech forests) | 17 |
| 20 (loess hills – oak-cerium forests) | 32 | 65 (rugged highlands on variegated Mesozoic rocks – beech-fir forests) | 3 |
| 21 (loess hills – oak forests) | 1 | 67 (rugged highlands or lower mountains on variegated rocks of Klippen belt – oak-hornbeam forests) | 4 |
| 22 (loess hills – oak-hornbeam forests) | 7 | 68 (rugged highlands or lower mountains on variegated rocks of Klippen belt – beech forests) | 2 |
| 23 (polygenic hilly or fragmented pediments – oak-cerium forests) | 15 | 69 (rugged highlands on crystalline rocks – oak-hornbeam forests) | 14 |
| 24 (polygenic hilly or fragmented pediments – oak forests) | 2 | 70 (rugged highlands on crystalline rocks – oak-beech forests) | 2 |
| 25 (polygenic hilly or fragmented pediments – oak-hornbeam forests) | 27 | 71 (rugged highlands on crystalline rocks – beech forests) | 10 |
| 26 (polygenic hilly or fragmented pediments – oak-beech forests) | 9 | 81 (karst mountain plain – beech forests) | 1 |
| 27 (polygenic hilly or fragmented pediments – beech forests) | 3 | 84 (rugged volcanic lower mountains – beech forests) | 9 |
| 28 (polygenic hilly or fragmented pediments – beech-fir forests) | 1 | 85 (rugged flysch lower mountains – beech forests) | 5 |
| 29 (polygenic hilly or fragmented pediments – fir-spruce forests) | 4 | 86 (rugged flysch lower mountains – beech-fir forests) | 3 |
| 31 (low plateau foothills – oak-cerium forests) | 10 | 87 (rugged flysch lower mountains – fir-spruce forests) | 1 |
| 32 (low plateau foothills – oak forests) | 1 | 89 (rugged flysch lower mountains – beech forests) | 3 |
| 33 (low plateau foothills – oak-hornbeam forests) | 30 | 90 (rugged karst lower mountains – beech-fir forests) | 1 |
| 34 (low plateau foothills – oak-beech forests) | 11 | 91 (rugged lower rock on crystalline rocks – oak-beech forests) | 4 |
| 35 (low plateau foothills – beech forests) | 10 | 92 (rugged lower rock on crystalline rocks – beech forests) | 7 |
| 39 (highland plain undifferentiated – beech forests) | 1 | 93 (rugged lower rock on crystalline rocks – beech-fir forests) | 3 |
| 40 (highland plain in volcanic highlands – oak-cerium forests) | 1 | 95 (very strongly rugged karst slope in lower mountains – beech-hornbeam forests) | 2 |
| 43 (karst highland plain – oak-cerium forests) | 1 | 97 (very strongly rugged karst slope in lower mountains – beech forests) | 2 |
| 45 (rugged volcanic highlands – oak-cerium forests) | 7 | 98 (very strongly rugged karst slope in lower mountains – beech-fir forests) | 7 |
| 46 (rugged volcanic highlands – oak forests) | 2 | 105 (very strongly rugged karst slopes in higher mountains – beech-fir forests) | 4 |
| 47 (rugged volcanic highlands – oak-hornbeam forests) | 15 | 105 (rugged higher mountains on variegated Mesozoic rocks – beech-fir forests) | 7 |
